# Development and psychometric properties of surveys to assess provider perspectives on the barriers and facilitators of effective care transitions

**DOI:** 10.1186/s12913-021-06369-5

**Published:** 2021-05-20

**Authors:** Maurice C. Johnson, Helen Liu, Joann Sorra, Jane Brock, Brianna Gass, Jing Li, Jessica Miller Clouser, Karen Hirschman, Deborah Carpenter, Huong Q. Nguyen, Mark V. Williams

**Affiliations:** 1 Westat, 1600 Research Boulevard, Rockville, MD USA; 2 Telligen Quality Improvement Organization, Denver, CO USA; 3 Center for Health Services Research, University of Kentucky, Lexington, KY USA; 4 University of Pennsylvania, Philadelphia, PA USA; 5 Kaiser Permanente Southern California, Pasadena, CA USA

**Keywords:** Care transitions, ACHIEVE, Psychometrics, Barriers, Facilitators, Transitional care, Provider experience, Composite measures, Health care surveys

## Abstract

**Background:**

The quality of the discharge process and effective care transitions between settings of care are critical to minimize gaps in patient care and reduce hospital readmissions. Few studies have explored which care transition components and strategies are most valuable to patients and providers. This study describes the development, pilot testing, and psychometric analysis of surveys designed to gain providers’ perspectives on current practices in delivering transitional care services.

**Methods:**

We underwent a comprehensive process to develop items measuring unique aspects of care transitions from the perspectives of the three types of providers (downstream, ambulatory, and hospital providers). The process involved 1) an environmental scan, 2) provider interviews, 3) survey cognitive testing, 4) pilot testing, 5) a Stakeholder Advisory Group, 6) a Scientific Advisory Council, and 7) a collaborative Project ACHIEVE (Achieving Patient-Centered Care and Optimized Health in Care Transitions by Evaluating the Value of Evidence) research team. Three surveys were developed and fielded to providers affiliated with 43 hospitals participating in Project ACHIEVE. Web-based survey administration resulted in 948 provider respondents. We assessed response variability and response missingness. To evaluate the composites’ psychometric properties, we examined intercorrelations of survey items, item factor loadings, model fit indices, internal consistency reliability, and intercorrelations between the composite measures and overall rating items.

**Results:**

Results from psychometric analyses of the three surveys provided support for five composite measures: 1) *Effort in Coordinating Patient Care*, 2) *Quality of Patient Information Received*, 3) *Organizational Support for Transitional Care*, 4) *Access to Community Resources*, and 5) *Strength of Relationships Among Community Providers*. All factor loadings and reliability estimates were acceptable (loadings ≥ 0.40, α ≥ 0.70), and the fit indices showed a good model fit. All composite measures positively and significantly correlated with the overall ratings (0.13 ≤ *r* ≤ 0.71).

**Conclusions:**

We determined that the items and composite measures assessing the barriers and facilitators to care transitions within this survey are reliable and demonstrate satisfactory psychometric properties. The instruments may be useful to healthcare organizations and researchers to assess the quality of care transitions and target areas of improvement across different provider settings.

**Supplementary Information:**

The online version contains supplementary material available at 10.1186/s12913-021-06369-5.

## Background

Since the Hospital Readmissions Reduction Program, enacted as part of the Patient Protection and Affordable Care Act (PPACA) [1], there has been an increase in efforts to improve care transitions from the hospital to another care settings [2, 3]. A variety of care transition initiatives aim to improve care continuum for patients between care settings while reducing non-beneficial hospital readmissions and improving health outcomes [4–8]. Studies have shown that multi-component care transition initiatives can effectively reduce all-cause readmissions for health care organizations, systems, and payers [9–13]. Few studies, however, have provided insight on what matters most to patients, caregivers, or care providers in meeting their needs in care transitions. As these individuals have direct interactions with the health care system, it is imperative that we understand which initiatives are essential in achieving successful outcomes and how to effectively implement these programs to improve the quality of care patients receive.

Project ACHIEVE (Achieving Patient-Centered Care and Optimized Health in Care Transitions by Evaluating the Value of Evidence) was a multi-stakeholder research project launched in 2015. The study applied a mixed-methods approach to defining patient-desired outcomes of care transitions, assessing practices that hospitals used to improve care transitions, and analyzing the relationship between care transition strategies and health outcomes [14, 15]. Another objective of Project ACHIEVE was to understand provider perspectives on the barriers and facilitators of effective care transitions [16]. Previous studies examining provider perspectives on care transitions and continuity generally surveyed a single representative from a provider organization [17–19] or administered the survey within a single care setting [20, 21] or health care system [22, 23]. The Project ACHIEVE Provider Surveys were developed to 1) gain providers’ perspectives on current practices in delivering transitional care services, 2) assess important barriers and facilitators in providing transitional care services, and 3) identify the organizational and community contexts that affect transitional care services from multiple care provider perspectives.

This paper outlines the Project ACHIEVE Provider Surveys’ development process. It discusses its prospective research design [14], highlights outcomes from its cognitive and pilot testing, and presents findings from the survey psychometric analyses, including factor structure, reliability, and composite measures.

## Methods

### Content and survey item development

The intended respondents of the ACHIEVE Provider Surveys were providers who coordinated the care of discharged patients from a participating hospital. We started identifying potential content areas through an environmental scan of known and ongoing efforts to improve care transitions through coordinated action among providers delivering services in different settings. A listing of the surveys identified in the environmental scan is presented in a separate file (See Supplement 1).

Concurrently, the research team conducted a series of key informant interviews with providers in the U.S. to explore factors that impacted the implementation of care transition programs in different settings. We recruited providers actively engaged in care transition efforts. Many of these providers were from the Centers for Medicare and Medicaid Services (CMS) Quality Improvement Organizations’ (QIO) *Integrate Care for Populations & Communities (ICPC) Aim* in the 10th Scope of Work and the *Community-based Care Transitions Program (CCTP)* [24, 25]. Interviewees represented providers from different healthcare settings, including hospitals, primary care offices, skilled nursing facilities, and long-term care facilities; and serving various roles within their institutions, including hospitalists, nurses, case managers, care navigators, clinicians, and staff. The research team conducted structured interviews with 63 representatives from 23 communities across 14 states. The topic areas included implementing barriers and facilitators to transitional care initiatives, the sustainability of initiatives, monitoring and evaluating initiatives, and community collaboration. Interviews were scheduled for 90 min to 120 min. Interviews were audio-recorded and coded using an iterative process; two research team members coded each interview independently and then met to resolve any coding discrepancies. Emergent themes were identified, and transcripts were further analyzed to determine if patterns existed among themes and various community characteristics (e.g., demographics, provider discipline, organizational affiliation, and type of care transitions improvement efforts). For example, the theme of challenges with communication and information exchange was identified among skilled nursing facilities, community physicians, and community-based organizations. Types of statements these providers made included “having to dig for information in a discharge summary” and “the format of communication received, or information that is accessible is problematic.”

The research team also regularly engaged its Stakeholder Advisory Group (SAG) and Scientific Advisory Council (SAC) meeting quarterly over 18 months. Each group included a broad spectrum of experts (e.g., patients, caregivers, clinicians, policymakers, advocacy groups, health professional associations, and other healthcare system stakeholders) tasked with advising the research team throughout the survey development process. The research team presented findings from the environmental scans and provider interviews, intending to identify and select the most salient topics to address in the ACHIEVE Provider Surveys. The research team was selective in the number of content areas and the number of questions the surveys would address to minimize providers’ burden. The ACHIEVE research team yielded eight main content areas for inclusion in the Provider Surveys through an iterative consensus process with the SAG and SAC. These content areas included:

1. **Effort in Coordinating Patient Care:** Provider assessment of their ability to obtain information about a recently discharged or soon to be discharged patient.
2. **Quality of Patient Information Received:** Provider assessment of the comprehensiveness of the information they received about a recently discharged patient or soon to be discharged patient.
3. **Organizational Support for Transitional Care:** Provider assessment of the provider’s organizational and senior leadership support for providing transitional care services.
4. **Access to Community Resources:** Provider assessment of patient access to services and health-related resources within their community.
5. **Strength of Relationships Among Community Providers:** Provider assessment of how the provider worked with other types of providers in the community when providing transitional care to patients.
6. **Receipt of Information from Hospital:** Provider assessment of timeliness of patient information from hospital and knowledge of how the provider learned about a patient’s admission and discharge from hospital.
7. **Communication with Caregivers:** Provider assessment of how often they interacted with a family or friend caregiver of a patient.
8. **Health Information Technology:** Information on whether providers had access to a hospital’s electronic medical record system.

While the findings and questionnaires identified from the environmental scan and the SAG/SAC provided insight on the main content to include in the surveys, each survey item in the Project ACHIEVE Provider Survey was originally developed.

### Cognitive testing

We completed two rounds of cognitive testing (July 28, 2015 – August 30, 2015; March 18, 2016 – April 15, 2016) with 28 total providers to pretest iterations of questions for the survey. The tests were generally 60 min and conducted via telephone. Providers were given an electronic version of the survey before the interview. During the cognitive interview, we asked respondents to discuss their thought process when answering survey items. The cognitive testing allowed the research team to examine how participants comprehended and processed the draft survey items and instructions, retrieved information from memory, formulated their responses, and chose their answers from a set of response options. Providers serving in care delivery (e.g., hospitalists, social workers, care coordinators, nurses, administrators, and primary care providers) participated in the cognitive testing. These providers represented a range of organizations, including acute care hospitals, primary care clinics, home healthcare agencies, and skilled nursing facilities.

Cognitive interviews were recorded and transcribed, with two research team members independently coding themes and meeting to discuss discrepancies. Cognitive testing revealed a need for more than one version of the survey, as the research team found not all questions were relevant to providers from different settings. We learned that providers perceived differing roles in delivering care to patients depending on if it was before or after a patient’s discharge from a hospital. Within a hospital setting, providers typically felt they had access to patients’ care plans during their time in the hospital, were responsible for patients’ care during a relatively short period, and were mainly focused on communicating with the hospital’s care discharge team. Conversely, primary care providers generally felt they had long-term knowledge of their patients and were reliant on the patient or hospital informing them about a hospital admission. Providers from skilled nursing facilities and home health agencies also felt reliant on a hospital or patient telling them about a patient’s time in the hospital. Generally, they did not have relationships with their patients before a hospital admission. We developed three provider surveys to accommodate unique features of setting-specific practices and relevance of activities for discharging versus receiving functions in transitional care:

1. **Downstream Provider:** Healthcare providers in skilled nursing facilities, home health agencies, or other community-based organizations that coordinated with hospitals to provide care to patients recently discharged from a hospital. These providers included intake coordinators, care coordinators, health coaches, or similar positions.
2. **Ambulatory Provider:** Primary care and specialty providers in ambulatory care settings who coordinated with hospitals to provide care to patients recently discharged from a hospital. These providers included physicians, physician assistants, and nurse practitioners who provided care to patients recently discharged from a hospital.
3. **Hospital Provider:** Providers involved with care or services that supported hospital discharge processes. These included case managers, care coordinators, nurses, physicians, physician assistants/nurse practitioners, pharmacists, or social workers.

Questions were tailored to each provider type. For example, the survey asked hospital providers about the quality of patient information they received *within the hospital*. In contrast, the survey asked downstream and ambulatory providers about the quality of patient information they received from a specific hospital.

### Measures

We used five-point response scales of agreement (“Strongly disagree” to “Strongly agree”), frequency (“Never” to “Always”), or rating (“Poor” to “Excellent”), for most times, and included a “Does not apply” or “Don’t know” response option for selected items. Many of the items were constituent to a composite measure – that is, an overall summary measure composed of two or more survey items that were closely related conceptually [26]. These composite measure constituent items typically shared the same question stem and response scale [27]. In addition to composite measure items, there were single-item measures, outcome ratings, and descriptive items in the survey.

#### Composite measures

We developed five predefined composite measures that were similar across all three Provider Surveys:

1. **Effort in Coordinating Patient Care** (3 or 4 items)^1^
2. **Quality of Patient Information Received** (4 items)
3. **Organizational Support for Transitional Care** (2 or 3 items)
4. **Access to Community Resources** (6 items)
5. **Strength of Relationships Among Community Providers** (4 items)

#### Single item measures and descriptive items

Three single-item measures were unique to the Downstream and Ambulatory Provider Surveys. These questions pertained to the discharge summaries providers received from specified hospitals. All three surveys had a single-item asking about provider’s interactions with patients’ family or friend caregivers. The surveys also contained a series of descriptive items capturing the providers’ characteristics and backgrounds (i.e., access to hospital electronic medical records, type of provider, years of delivering care to patients).

#### Overall rating measures

We developed two overall rating measures to assess how well a specified hospital coordinated with downstream and ambulatory providers in delivering care to patients and determine how well an organization provided transitional care to patients:

1. **Rating of Coordination with a Hospital**: *Please rate how well the hospital coordinates with you when working with recently discharged patients (Poor/Fair/Good/Very Good/Excellent)*
2. **Rating of Organization’s Delivery of Transitional Care to Patients**: *Please rate how well your organization helps transition patients from the hospital to another healthcare setting or back home (Poor/Fair/Good/Very Good/Excellent)*

### Pilot testing

A small pilot test was conducted before full-scale data collection, using REDCap (Research Electronic Data Capture) [28], to collect and manage web-based surveys during an 11-week period in 2017. A total of 110 respondents (21 downstream providers, 30 ambulatory providers, and 59 hospital providers) from 5 ACHIEVE hospitals participated in the pilot. The pilot aimed to test the usability and functionality of web surveys, reduce duplicative questions, and identify survey items that needed further refinement. The pilot results led to the removal of 6 questions and the rewording of 13 individual items to streamline the surveys for the primary data collection.

### Main data collection

Forty-three Project ACHIEVE hospitals participated in the Provider Surveys’ primary data collection, representing various regions across the U.S. and different types of hospitals (e.g., non-government non-profit, teaching institutions, and integrated delivery systems). The ACHIEVE Provider Surveys administered during the study’s primary data collection phase are presented in a separate file (See Supplement 2). Compared to the American Hospital Association (AHA) short-term acute care/critical access hospitals (*N* = 4700), Project ACHIEVE hospitals (*N* = 43) were primarily non-government non-profit (59% AHA vs. 81% ACHIEVE), large hospitals with at least 300 licensed beds (19% AHA vs. 53% ACHIEVE), and from the West (20% AHA vs. 40% ACHIEVE).

An ACHIEVE coordinator at each hospital was responsible for recruiting providers for each of the three surveys. Recruitment started from participating hospitals and communities and targeted providers familiar or actively involved with transitional care efforts. Each participating hospital nominated relevant hospital and downstream providers based on whether they were actively involved in care transitions efforts. The study intended to have completed Hospital Surveys from at least one in-patient physician, floor nurse, case manager, and social worker from each participating hospital. The study targeted 1–2 intake coordinators, care coordinators, or similar positions from each partnering SNF, HHA, and CBO for the Downstream Provider survey; the study’s goal was to have nearly four SNFs and HHAs per participating hospital and completed surveys for about 22 CBOs in total. Last, for the Ambulatory Provider Survey, we focused recruitment to the top referring community physicians for each participating hospital and estimated eight providers per hospital.

REDCap web-based surveys were administered by email using a generic public link (not customized to each provider) from November 2017 to April 2018. Since it was not possible to precisely determine how many providers received an invitation, we could not calculate response rates to the survey. The study offered a $30 incentive. All participants submitted written or oral informed consent. IRBs at the University of Kentucky, Kaiser Permanente, and Westat approved the study protocol.

### Analyses

We assessed the survey psychometric properties by examining item response variability and missing data, intercorrelations of the items, factor loadings and model fit, internal consistency reliability, and intercorrelations of the composite measures with the overall rating measures.

#### Analysis dataset

After removing ineligible responses (i.e., providers not affiliated with the ACHIEVE hospitals or did not answer any substantive questions in the survey), the analytic dataset had 948 records: 381 downstream providers recruited from 40 hospitals, 284 ambulatory providers from 30 hospitals, and 283 hospital providers from 39 hospitals. Table 1 displays the characteristics of the study respondents. Physicians, physician assistants, nurse practitioners, and nurses combined made up the largest provider category among the Hospital (39%) and Ambulatory (83%) Provider Survey respondents. In comparison, the largest provider category among the Downstream Provider Survey respondents were administrators/managers (37%). Nearly half of the Hospital (47%) and Downstream (45%) Provider Survey respondents worked 1 to 5 years in their organization’s current role. In contrast, most Ambulatory Provider Survey respondents worked 6 or more years (72%).

**Table 1:** Characteristics of Study Respondents

Provider Characteristics	Downstream Provider Survey Respondents		Ambulatory Provider Survey Respondents		Hospital Provider Survey Respondents	
	N	%	N	%	N	%
**Total Respondents (% of 948 providers)**	381	40%	284	30%	283	30%
**Position within organization**						
Administrator/Manager	142	37%	--	--	48	17%
Physician	41	11%	189	67%	70	25%
Physician Assistant/Nurse Practitioner/Advance Practice Nurse	13	3%	45	16%	10	4%
Nurse	33	9%	--	--	28	10%
Care Coordinator/Case manager	59	16%	--	--	52	18%
Other	93	24%	50	18%	75	27%
**Time in current role**						
Less than 1 year	48	13%	19	7%	29	10%
1 to 5 years	173	45%	55	19%	134	47%
6 or more years	156	41%	204	72%	118	42%
Missing	4	0%	6	0%	2	0%

Note: Percent totals may not sum to exactly 100 percent due to rounding

#### Item analysis and inter-item correlations

As the first step in the analysis, we examined item frequencies to review the variability of responses. Survey items were flagged if they had 1) low variability (e.g., more than 90% of responses answered positively), or 2) a significant percentage of missing or “Does not apply or don’t know” responses (i.e., Missing/NA/DK 30% or higher). We also examined the intercorrelations of survey items to determine how strongly the items were related. Ideally, items hypothesized to measure the same construct should relate to one another with moderate or moderately high correlations. However, excessively high correlations – for example, above 0.90 – suggest a significant overlap or redundancy in content. On the other hand, very low correlations signal weak relationships among items.

#### Confirmatory factor analysis (CFA)

We conducted a CFA to assess whether the items proposed for the five composite measures adequately loaded on the factors or composites they were intended to measure [29]. The criterion for factor loadings of 0.40 or greater was used to indicate that the item’s relationship to the composite measure was acceptable [30]. We also examined overall model fit statistics: the chi-square, comparative fit index (CFI), the root mean square error of approximation (RMSEA), and the standardized root mean square residual (SRMR). We examined the chi-square index divided by the degrees of freedom, using a criterion of less than 5.00 [31]. The CFI compares the existing model fit with a null model that assumes the latent variables in the model are uncorrelated. The factor structure is considered an adequate fit to the data if the CFI is at least 0.95 [32]. The RMSEA is a parsimony-adjusted index that favors the simplest model possible [33]. A RMSEA less than 0.06 is considered good fit [32]. The SRMR is the standardized difference between the observed covariance and predicted covariance. A value of zero for the SRMR indicates perfect fit, but a value less than 0.08 is considered good fit [34].

#### Internal consistency reliability

We conducted reliability analyses on the final composite measures to ensure that individuals responded consistently to the items within each composite. We examined internal consistency by calculating Cronbach’s alpha for each of the composites to assess the extent to which respondents answered consistently to the theoretically similar items in each composite. Cronbach’s alpha (α) ranges from 0 to 1.00, with higher alphas indicating better reliability. The minimum criterion for acceptable reliability is an alpha of at least 0.70 [35]. We also examined the impact of deleting one of the items on alpha.

#### Intercorrelations of composite measures and overall ratings

As the final analysis step, we examined the relationships between the final composite measures and the hospital’s and organization’s overall ratings on care transitions using Spearman rank-order correlations. Very high intercorrelations (e.g., > 0.80) indicate that the composite measures may not be unique enough to be considered separate measures. In contrast, very low intercorrelations would suggest that the measures are not related. We hypothesized that the composites would have positive, moderate to strong intercorrelations with the overall rating items. All analyses were completed using SAS 9.4.

## Results

### Item analysis and inter-item correlations

Most items across the three surveys showed adequate variability, except item 19 - *Reducing hospital readmissions for patients is a priority in my organization* – where over 90% of respondents (95% downstream, 93% ambulatory, and 91% hospital) answered Strongly Agree or Agree. This suggested that reducing readmissions was generally perceived as a priority by almost all providers. Therefore, we decided to drop this item from the composite analysis. However, we kept it as a single-item measure because our SAG and SAC considered the item contextually important and potentially useful for comparisons outside of Project ACHIEVE’s study population. Furthermore, no items had more than 30% missing when combining Missing/NA/DK. The combined percent missing ranged up to 5% for the Downstream Provider, up to 6% for the Ambulatory Provider, and up to 7% for the Hospital Provider Survey. An additional file provides summary responses (See Supplement 3).

We also examined the intercorrelations of items comprising each of the five proposed composite measures. All five composite measure constituent items were significantly interrelated with correlations ranging from 0.12 to 0.72 for the Downstream, 0.31 to 0.74 for the Ambulatory, and 0.22 to 0.79 for the Hospital Provider Survey. Item 9 from the Downstream Provider Survey - *Too many of the patients referred to our services have more acute conditions than we are able to handle* - had small correlations with the other three items in the proposed composite measure *Effort in Coordinating Patient Care* (ranging from 0.12 to 0.18). Because the item provided descriptive information but did not fit well in a composite, we kept item 9 as a single-item measure.

#### Confirmatory factor analysis (CFA)

Table 2 presents standardized factor loadings of the items comprising the five composite measures. All factor loadings were statistically significant (*p* < 0.05) with magnitudes greater than or equal to 0.40, indicating that the items adequately loaded on the respective composite measures. Standardized factor loadings ranged from 0.54 to 0.90 for Downstream Provider, 0.63 to 0.95 for Ambulatory Provider, and 0.48 to 0.90 for Hospital Provider Survey.

**Table 2:** Confirmatory Factor Analysis Results by Provider Type

Composite Measures and Items		Factor Loading		
		Downstream	Ambulatory	Hospital
**Effort in Coordinating Patient Care**				
Q6	It is easy to get information about a recently discharged patient.	0.76	0.79	0.66
Q7	It is easy to connect with providers and staff in the hospital to discuss a patient’s care.	0.73	0.73	0.83
Q8	It is clear what in-patient procedures and tests have been performed and the results.	0.86	0.85	0.80
Q10	Everyone involved in the patient’s care understands what needs to be done for the patient.	–	–	0.84
**Quality of Patient Information Received**				
For recently discharged patients, how often is the information you receive:				
Q11	In a format where it is easy to find important information?	0.84	0.87	0.81
Q12	Complete?	0.82	0.88	0.82
Q13	Available as soon as it is needed?	0.84	0.87	0.79
Q14	Clear about who to follow up with at the hospital if you have questions or concerns about the patient?	0.73	0.80	0.83
**Organizational Support for Transitional Care**				
Q17	My organization is implementing activities to improve transitional care for patients.	0.81	0.65	0.66
Q18	Senior leaders in my organization dedicate adequate resources to support effective transitional care for patients.	0.89	0.95	0.88
Q20	My organization tries to increase physician awareness and understanding of the services we provide that can assist recently discharged patients.	0.81	–	–
**Access to Community Resources**				
In the local area your organization serves, patients have adequate access to:				
Q21	Primary care providers.	0.76	0.65	0.75
Q22	Specialty providers.	0.79	0.70	0.76
Q23	Skilled nursing and rehabilitation facilities.	0.70	0.75	0.66
Q24	Mental health/behavioral health services.	0.63	0.70	0.59
Q25	In-home support services (e.g., home health aides/ technicians or other services that help patients remain in their homes).	0.69	0.76	0.59
Q26	Transportation for medical-related services.	0.60	0.67	0.48
**Strength of Relationships Among Community Providers**				
How would you describe the relationship between you and the following providers in working together to provide transitional care to patients?				
Q27	Primary care providers and specialists.	0.54	0.63	0.68
Q28	Skilled nursing and rehabilitation facilities.	0.78	0.90	0.89
Q29	Home health agencies.	0.90	0.88	0.90
Q30	Community-based organizations.	0.74	0.80	0.80

All factor loadings were statistically significant at *p* < 0.05

Table 3 presents the model fit indices for the five-composite model. The comparative fit indices (CFI) for the Downstream, Ambulatory, and Hospital Provider Surveys were 0.95, 0.94, and 0.91, respectively, with CFIs for the Ambulatory and Hospital Provider Surveys slightly below the criterion (≥ 0.95). The SRMR scores showed a good fit (< 0.08), but the RMSEAs were marginally higher than the acceptable criterion (< 0.06) for all three surveys. Finally, the relative chi-square values (chi-square value divided by the degrees for freedom) for the Downstream, Ambulatory, and Hospital Provider Surveys were 1.91, 2.02, and 2.16, respectively, all meeting the criterion (< 5.00).

**Table 3:** Confirmatory Model Fit Indices by Provider Type

Confirmatory Factor Analysis Fit Indices						
Provider Type	***χ***^**2**^	***df***	***χ***^**2**^/***df***	CFI	RMSEA (90% CI)	SRMR
Downstream	295.95	155	1.91	0.95	0.06 (0.05–0.07)	0.05
Ambulatory	276.63	137	2.02	0.94	0.07 (0.06–0.09)	0.05
Hospital	335.55	155	2.16	0.91	0.08 (0.07–0.09)	0.07

χ^2^ chi-square (*p* < 0.05), *df* degrees of freedom, χ ^2^/ *df* relative chi-square (criterion < 5.00)

*CFI* comparative fit index (criterion ≥ 0.95), *RMSEA* root mean square error of approximation (criterion < 0.06)

*SRMR* standardized root mean square residual (criterion < 0.08), *CI* confidence interval

#### Internal consistency reliability

As presented in Table 4, the reliability for all five composite measures for each of the three surveys exceeded the 0.70 Cronbach’s alpha criterion, ranging from 0.82 to 0.88 for the Downstream, 0.79 to 0.89 for the Ambulatory, and 0.72 to 0.87 for the Hospital Provider Survey. The reliability statistics shown on the item rows indicate the composite’s reliability if that item was deleted. For all three survey versions, item 27 from the *Strength of Relationships Among Community Providers* composite would increase the composite’s reliability if deleted. However, since the increase in alpha was minimal, we decided to keep the item in the composite because it adds meaningful information.

**Table 4:** Final Reliability Analysis Results by Provider Type

Composite Measures and Items		Reliability if item deleted (Composite measure reliability)		
		Downstream	Ambulatory	Hospital
**Effort in Coordinating Patient Care**		(0.82)	(0.82)	(0.81)
Q6	It is easy to get information about a recently discharged patient.	0.75	0.74	0.81
Q7	It is easy to connect with providers and staff in the hospital to discuss a patient’s care.	0.76	0.83	0.74
Q8	It is clear what in-patient procedures and tests have been performed and the results.	0.73	0.67	0.73
Q10	Everyone involved in the patient’s care understands what needs to be done for the patient.	–	–	0.77
**Quality of Patient Information Received**		(0.86)	(0.89)	(0.86)
For recently discharged patients, how often is the information you receive:				
Q11	In a format where it is easy to find important information?	0.82	0.84	0.84
Q12	Complete?	0.81	0.83	0.80
Q13	Available as soon as it is needed?	0.82	0.84	0.83
Q14	Clear about who to follow up with at the hospital if you have questions or concerns about the patient?	0.85	0.89	0.82
**Organizational Support for Transitional Care**		(0.88)	(0.79)	(0.72)
Q17	My organization is implementing activities to improve transitional care for patients.	0.85	–	–
Q18	Senior leaders in my organization dedicate adequate resources to support effective transitional care for patients.	0.80	–	–
Q20	My organization tries to increase physician awareness and understanding of the services we provide that can assist recently discharged patients.	0.84	–	–
**Access to Community Resources**		(0.84)	(0.85)	(0.81)
In the local area your organization serves, patients have adequate access to:				
Q21	Primary care providers.	0.81	0.83	0.78
Q22	Specialty providers.	0.80	0.82	0.77
Q23	Skilled nursing and rehabilitation facilities.	0.81	0.81	0.79
Q24	Mental health/behavioral health services.	0.82	0.82	0.78
Q25	In-home support services (e.g., home health aides/ technicians or other services that help patients remain in their homes).	0.81	0.82	0.79
Q26	Transportation for medical-related services.	0.83	0.84	0.80
**Strength of Relationships Among Community Providers**		(0.82)	(0.87)	(0.87)
How would you describe the relationship between you and the following providers in working together to provide transitional care to patients?				
Q27	Primary care providers and specialists.	0.83	0.89	0.88
Q28	Skilled nursing and rehabilitation facilities.	0.75	0.80	0.80
Q29	Home health agencies.	0.74	0.80	0.81
Q30	Community-based organizations.	0.79	0.83	0.83

#### Intercorrelations among composite measures and overall ratings

Table 5 presents the intercorrelations of the composite measures and the overall ratings of *Coordination with a Hospital* and the *Organization’s Delivery of Transitional Care to Patients* for each of the three Provider Surveys. All correlations were statistically significant (*p* < 0.05), with magnitudes ranging from 0.13 to 0.63 for the Downstream, 0.33 to 0.71 for the Ambulatory, and 0.33 to 0.63 for the Hospital Provider Survey. The highest intercorrelations among the composite measures for all three provider types were between *Effort in Coordinating Patient Care* and *Quality of Patient Information Received*.

**Table 5:** Intercorrelations of the Composites and Overall Ratings by Provider Type

Composites and Overall Ratings	Provider Type	(1)	(2)	(3)	(4)	(5)	(6)	(7)
**Composite Measures**								
(1) Effort in Coordinating Patient Care	Downstream	–						
	Ambulatory	–						
	Hospital	–						
(2) Quality of Patient Information Received	Downstream	0.63	–					
	Ambulatory	0.71	–					
	Hospital	0.63	–					
(3) Organizational Support for Transitional Care	Downstream	0.13	0.21	–				
	Ambulatory	0.39	0.36	–				
	Hospital	0.47	0.47	–				
(4) Access to Community Resources	Downstream	0.14	0.22	0.34	–			
	Ambulatory	0.38	0.43	0.35	–			
	Hospital	0.33	0.35	0.35	–			
(5) Strength of Relationships Among Community Providers	Downstream	0.14	0.21	0.42	0.41	–		
	Ambulatory	0.33	0.49	0.36	0.54	–		
	Hospital	0.46	0.50	0.45	0.43	–		
**Overall Ratings**								
(6) Rating of Coordination with a Hospital	Downstream	0.47	0.51	0.30	0.27	0.50	–	
	Ambulatory	0.50	0.59	0.42	0.40	0.42	–	
	Hospital	–	–	–	–	–	–	
(7) Rating of Organization’s Delivery of Transitional Care to Patients	Downstream	0.24	0.26	0.44	0.32	0.56	0.55	–
	Ambulatory	0.42	0.48	0.58	0.35	0.39	0.63	–
	Hospital	0.51	0.51	0.54	0.45	0.67	–	–

All correlations were statistically significant at *p* < 0.05

Additionally, all five composite measures were significantly related to the two Overall Ratings for the Downstream and Ambulatory Provider Surveys (note that the Hospital Provider Survey did not ask about *Coordination with a Hospital*). The correlations between the five composite measures and the overall rating of *Coordination with a Hospital* ranged from 0.27 to 0.51 for the Downstream Providers and 0.40 to 0.59 for the Ambulatory Providers. The highest correlations in both surveys were with the *Quality of Patient Information Received*.

The correlations between the five composite measures and the overall rating of the *Organization’s Delivery of Transitional Care to Patients* ranged from 0.24 to 0.56 for the Downstream, 0.35 to 0.58 for the Ambulatory, and 0.45 to 0.67 for the Hospital Providers. The highest correlations were with *Strength of Relationships Among Community Providers* for both the Downstream and the Hospital Providers. In contrast, for the Ambulatory Providers, the highest correlation was with *Organizational Support for Care Transitions*.

## Discussion

The ACHIEVE Provider Surveys provided reliable measures to assess provider experiences and perspectives of the barriers and facilitators in care transitions. Our surveys targeted three types of providers – downstream, ambulatory, and hospital, and measured unique aspects of care transition efforts that were key to ensuring patient care continuity from one setting to another. We developed five composite measures encompassing core components of care transitions based on providers’ real-world experiences in improvement initiatives. The surveys consisted of descriptive items, multi-item composite measures, single-item measures, and overall ratings (total number of final survey items – 36 for Downstream, 36 for Ambulatory, and 27 for Hospital Provider Survey). The CFA and reliability analysis for the five-composite models showed a good model fit to the data and indicated that each survey item aligned with its respective measure construct. All factor loadings were statistically significant (*p* < 0.05) with magnitudes ≥ 0.40, indicating that the constituent items adequately loaded on each of the five composite measures.

The associations among the five composite measures also provided support for their construct validity. For all three provider types, the strongest positive relationships were between *Effort in Coordinating Patient Care* and *Quality of Patient Information Received* (r = 0.63 for Downstream, r = 0.71 for Ambulatory, r = 0.63 for Hospital). Literature has shown that incomplete transfer of information and the absence of a healthcare professional who oversees care continuity can contribute to gaps in care during critical transitions [36]. Relatedly, a common theme that arose from the stakeholder interviews was that providers valued clear and effective communications about patient information.

We also explored relationships between the five composite measures and the two overall ratings --*Coordination with a Hospital* and *Organization’s Delivery of Transitional Care to Patients* -- to determine whether the composites were related to these self-reported outcomes. For both downstream and ambulatory providers, higher ratings of *Coordination with a Hospital* were associated with better *Quality of Patient Information Received*. This finding suggests that improvement in patient discharge information (e.g., clear and concise format, completeness, available promptly) may effectively facilitate the care transition of patients from a hospital [3, 37–39]. The *Organization’s Delivery of Transitional Care to Patients* was also found to have a strong positive association with *Strength of Relationships Among Community Providers* among downstream (e.g., SNFs and HHAs) and hospital providers. This relationship, however, was weaker among ambulatory providers (e.g., PCPs and specialists). This association suggests that while building provider relationships within a community can lead to better care transition outcomes, the impact may be more substantial among hospitals, SNFs, HHAs, and CBO’s than among primary and specialty care practices. It could be argued that ambulatory providers have limited resources and time to invest in collaborative efforts [40], and that the efforts may be dictated by the partnership role ambulatory providers have, especially if primary and specialty providers play a larger role as the “integrator” in health system models [41]. The differences in the perceived outcomes may also relate to the characteristics of the sample. For example, physicians, physician assistants, nurse practitioners, and advance practice nurses made up 14% of respondents of the Downstream Provider Survey vs. 83% of the Ambulatory Provider Survey.

### Limitations

Project ACHIEVE recruited hospitals to ensure representation of various hospitals and community-based organizations with regard to 1) urban and rural location, 2) safety-net and critical access status, 3) integrated delivery system participation, and 4) involvement in care delivery demonstrations (e.g., accountable care organization, bundled payments for care improvement) [14]. Despite this effort, the potential for self-selection bias of hospitals participating in Project ACHIEVE remains, and there could have been systematic differences between participants and non-participants. The hospitals that agreed to participate may be more actively addressing transitional care issues than non-participants. Similarly, providers recruited and agreed to participate may be over- or under-representative of particular positions or roles, and therefore their opinions may not reflect the perspectives of average providers. Future studies could recruit providers who are representative of the staff positions within organizations and levels of engagement with integrated transitional care programs to address this limitation. It would also be beneficial to investigate how the Provider Survey’s composite measures relate to patient outcomes, including those in the Project ACHIEVE patient and caregiver surveys [42], and whether top-performing hospitals in these measures would affect readmission rates or patient satisfaction with care. Finally, while we determined the instruments to be psychometrically sound, additional analyses could help compare the relative strength of the relationship between the proposed measures across care settings.

Taking into account the study’s limitations, the ACHIEVE Provider Surveys can serve as a tool to assess provider perspectives on how well healthcare organizations are currently delivering transitional care and track improvement over time. Specifically, the surveys assess patient information coordination among providers within and outside of a hospital and provide a framework for understanding the hospitals’ organizational and community environments and contexts. By evaluating three provider types, the survey may also help target quality improvement strategies to specific types of providers and settings of care.

## Conclusions

This study identified five conceptual domains of care transitions and developed three versions of an instrument that could serve as a set of leading indicators that reliably measure provider perspectives. These domains can potentially be used to assess the effectiveness of transitional care delivered by hospitals.

Our surveys were tailored to providers in three organizational settings typically engaged in care transitions improvement efforts: downstream, ambulatory, and hospital. Among the hospitals participating in Project ACHIEVE, the composite measures and individual items assessing barriers and facilitators to care transitions were overall psychometrically sound. The provider surveys may be useful to healthcare organizations and researchers aiming to assess the quality of care transitions and target areas of improvement across different types of providers and settings.

## Supplementary Information

**Additional file 1.** Environmental Scan Findings. Surveys and Questionnaires Identified from Project ACHIEVE Provider Survey Environmental Scan. A listing of surveys and questionnaires identified from the Project ACHIEVE environmental scan of provider surveys about the continuity of care and care transitions.

**Additional file 2.** ACHIEVE Provider Survey. ACHIEVE Downstream Provider, Ambulatory Provider, and Hospital Provider Surveys to assess transitional care. A set of surveys to be administered to providers to learn about their work experiences delivering transitional care to patients. Developed by Westat, the Provider Surveys’ goal is to assess the barriers and facilitators in delivering transitional care services and describe the organizational and community contexts in providing transitional care services.

**Additional file 3.** Summary of Responses Table. Summary of Responses Table-Mean, Standard Deviation, Percent Positive, Missing, Does Not Apply/Don’t Know Results. The table includes the mean, standard deviation, percent positive, missing, does not apply/don’t know results of each survey item by provider type.

## Data Availability

The datasets used and analyzed during the current study are available from the corresponding author on reasonable request.

## Abbreviations

ACHIEVE: Achieving patient-centered care and optimized health in care transitions by evaluating the value of evidence
CFA: Confirmatory factor analysis
CFI: Comparative fit index
RMSEA: Root mean square error of approximation
SAC: Scientific advisory council
SAG: Stakeholder advisory group
SRMR: Standardized root mean square residual

## Footnotes

1 Number of constituent items within the proposed composites depended on whether the item was asked in each of the three surveys (downstream, ambulatory, or hospital).
