# Supplementary material for: Development and psychometric properties of surveys to assess provider perspectives on the barriers and facilitators of effective care transitions
Source: BMC Health Serv Res. 2021 May 20;21:478. doi: 10.1186/s12913-021-06369-5 (PMC8136156; doi:10.1186/s12913-021-06369-5)
Supplement: Supplementary file 1 — Additional file 1. Environmental Scan Findings. Surveys and Questionnaires Identified from Project ACHIEVE Provider Survey Environmental Scan. A listing of surveys and questionnaires identified from the Project ACHIEVE environmental scan of provider surveys about the continuity of care and care transitions. [file 12913_2021_6369_MOESM1_ESM.pdf]

TITLE PAGE

Title: Development and psychometric properties of surveys to assess provider perspectives on the barriers and facilitators of effective care transitions

Supplement 1: Surveys and Questionnaires Identified from Project ACHIEVE Provider Survey Environmental Scan

Authors
Maurice C. Johnson, Jr., MPH[1]
Helen Liu, BS[1]
Joann Sorra, PhD[1]
Jane Brock, MD, MSPH[2]
Brianna Gass, MPH[2]
Jing Li, MD, DrPH, MS[3]
Jessica Miller Clouser, MPH[3]
Karen Hirschman, PhD, MSW[4]
Deborah Carpenter, RN, MSN[1]
Huong Q, Nguyen, PhD, RN[5]
Mark V. Williams, MD, MHM[3]

[1] Westat, Rockville, MD
[2] Telligen Quality Improvement Organization, Denver, CO
[3] Center for Health Services Research, University of Kentucky, Lexington, KY
[4] University of Pennsylvania, Philadelphia, PA
[5] Kaiser Permanente Southern California, Pasadena, CA

Corresponding Author
Maurice C. Johnson, MPH
Westat
1600 Research Boulevard, RB 1189
mauricejohnson@westat.com
240-453-2640

	Questionnaire/Study Name	Summary/Topic Areas Discussed	Citation
1	**American Academy of Pediatrics - Transitions**	Transition practices from youth to adult care, transition policy awareness, and transitional care barriers and needs.	Chira P, Ronis T, Ardoin S, White P. Transitioning youth with rheumatic conditions: perspectives of pediatric rheumatology providers in the United States and Canada. J Rheumatol 2014;41:768-779.
2	**American Society of Health-System Pharmacists Survey**	A national assessment survey of 393 pharmacy directors was conducted to assess pharmacist roles in transition-of care (TOC) activities in the United State. Topics include 1. Medication Reconciliation, 2. Admission histories, 3. Medication counseling, 4. Postdischarge follow-up, 5. Pharmacy student and pharmacy technician involvement in TOC activities	Kern, Kristine A., et al. "Variations in pharmacy-based transition-of-care activities in the United States: A national survey." American Journal of Health-System Pharmacy 71.8 (2014): 648-656.
3	**Assessment of Chronic Illness Care**	The content of the ACIC was derived from specific evidence-based interventions for the six components of the Chronic Care Model (community resources, health organization, self-management support, delivery system design, decision support and clinical information systems). Like the Chronic Care Model, the ACIC addresses the basic elements for improving chronic illness care at the community, organization, practice and patient level	Bonomi, A. E., Wagner, E. H., Glasgow, R. E., & VonKorff, M. (2002). Assessment of chronic illness care (ACIC): a practical tool to measure quality improvement. Health services research, 37(3), 791-820.
4	**CAHPS Clinician & Group Surveys**	Asks patients to report on their experiences with providers and staff in primary care and specialty care settings. Survey results can be used to: Determine the need for improvement activities and evaluate the impact of those efforts, and equip consumers with information they can use to choose physicians and other health care providers, physician practices, or medical groups.	CAHPS Clinician & Group Survey. Content last reviewed December 2020. Agency for Healthcare Research and Quality, Rockville, MD. https://www.ahrq.gov/cahps/surveys-guidance/cg/index.html
5	**California Healthcare Foundation Post-Hospital Follow-Up Visit**	Checklist based on extensive research and interviews with clinicians, offers a proposed checklist to help guide the encounter. It is divided into three sections: prior to the visit, during the visit, and at the conclusion of the visit.	Coleman, E. The Post-Hospital Follow-Up Visit: A Physician Checklist to Reduce Readmissions. California Health Care Foundation. October 2010. https://www.chcf.org/wp-content/uploads/2017/12/PDF-PostHospitalFollowUpVisit.pdf
6	**Care Transitions Innovation (C-TraIn) Survey**	In-depth interviews with participants prior to initiating a hospital-funded, multi-component transitional care intervention for uninsured and low-income publicly insured patients.	Davis, Melinda M., et al. ""Did I do as best as the system would let me?" Healthcare professional views on hospital to home care transitions." Journal of general internal medicine 27.12 (2012): 1649-1656.
7	**Clinician Support Patient Activation (CS-PAM)**	Measures clinician attitudes about the patient role in the care process.	Hibbard JH, Collins PA, Mahoney E, Baker LH. The development and testing of a measure assessing clinician beliefs about patient self-management. Health Expect. 2010 Mar;13(1):65-72. doi: 10.1111/j.1369-7625.2009.00571.x. Epub 2009 Nov 10. PMID: 19906211; PMCID: PMC5060511.
8	**Community Coalition Assessment Survey**	Informs ongoing coalition improvement, membership recruitment and leadership development. Areas include 1. Coalition Performance , 2. Coalition Synergy, 3. Coalition Leadership, 4. Coalition Coordination and Administration, 5. Coalition Decision-Making, 6. Coalition Capacity	Foster-Fishman, P.G., Berkowitz, S.L., Lounsbury, D.W., Jacobson, S. and Allen, N.A. Building Collaborative Capacity in Community Coalitions: A Review and Integrative Framework, American Journal of Community Psychology, Vol. 29, No. 2, 2001 https://www.mihealthtools.org/ccat/pdfs/CCATSurvey_PrintVersion.pdf

9	**Continuity of Care Instruments**	Review of instruments measuring continuity of care and to assess the quality of their measurement properties. The threedimensions of continuity of care include (1) care from the same provider who knows and follows the patient (personal continuity), (2) communication and cooperation between care providers in one care setting (team continuity), and (3) communication and cooperation between care providers in different care settings (cross-boundary continuity).	Uijen AA, Heinst CW, Schellevis FG et al. Measurement properties of questionnaires measuring continuity of care: a systematic review. PLoS One 2012;7:e42256.
#	**Council of Emergency Medicine Residency Directors Transitions of Care Task Force Needs Assessment**	Assesses current handoff practices and asked participants to rate the importance of key logistical and informational parameters within a transitions of care team.	Kessler, Chad, et al. "An algorithm for transition of care in the emergency department." Academic Emergency Medicine 20.6 (2013): 605-610.
#	**Critical Care Nurses Discharge Planning Process Survey**	To report on the beliefs of critical care nurses with regard to the discharge planning process. Areas discussed include personal and professional characteristics of nurse and discharge planning experiences	Watts R, Gardner H, Pierson J. Factors that enhance or impede critical care nurses' discharge planning practices. Intensive Crit Care Nurs. 2005 Oct;21(5):302-13. doi: 10.1016/j.iccn.2005.01.005. PMID: 16182126.
#	**Critical Intensive Care Units Survey**	Study survey to assess nurses awareness of patient transition experience, and nurses awareness of transisitonal care practice	Ludin, Salizar Mohamed, Steve Parker, and Paul Arbon. "A survey of Malaysian Critical Intensive Care unit nurses' awareness of patients' transition experiences (PE) and transitional care practice (TCP)." Intensive and Critical Care Nursing 30.4 (2014): 196-203.
#	**Discharging and Readmission Team Surveys**	Determine physician awareness of readmissions and the frequency, predictors, and content of physician communication regarding readmissions.	Roy CL, Kachalia A, Woolf S, Burdick E, Karson A, Gandhi TK. Hospital readmissions: Physician awareness and communication practices. Journal of General Internal Medicine 2009;24:374-380.
#	**Experience of Care Transitions**	A qualitative descriptive design was used to solicit patients', caregivers', and health-care providers' perceptions of care transitions, their role within the process, barriers to effective care transitions, and strategies to overcome these barriers.	Fuji KT, Abbott AA, Norris JF. Exploring care transitions from patient, caregiver, and health-care provider perspectives. Clin Nurs Res 2013;22:258-274.
#	**Framework for Understanding Care Transitions**	A descriptive framework illustrating the interconnected roles of patients, providers, and caregivers in relation to readmissions. 5 main themes emphasizing the necessity of a coordinated support network: (1) teamwork, (2) health systems navigation and management, (3) illness severity and health needs, (4) psychosocial stability, and (5) medications	Lee JI, Cutugno C, Pickering SP et al. The patient care circle: a descriptive framework for understanding care transitions. J Hosp Med 2013;8:619-626.
#	**General Practicioner - Netherlands**	Cross-sectional survey exploring a) the discharge of breast cancer patients to primary care by specialists, at the end of hospital follow-up and b) the experiences and views of general practitioners (GPs) regarding transfer of follow-up to the primary care setting	Roorda C, Berendsen AJ, Haverkamp M, van der Meer K, de Bock GH. Discharge of breast cancer patients to primary care at the end of hospital follow-up: a cross-sectional survey. Eur J Cancer 2013;49:1836-1844.

#	**Get With the Guidelines-Heart Failure Survey**	Survey of hospitals participating in the Get With the Guidelines-Heart Failure quality improvement program regarding common processes of care aimed at reducing readmissions. Processes grouped into 3 domains (ie, inpatient care, discharge and transitional care, and general quality improvement)	Kociol, Robb D., et al. "National survey of hospital strategies to reduce heart failure readmissions findings from the get with the guidelines-heart failure registry." Circulation: Heart Failure 5.6 (2012): 680-687. https://www.ahajournals.org/doi/10.1161/CIRCHEARTFAILURE.112.967406?url_ver=Z39.88-2003&rfr_id=ori:rid:crossref.org&rfr_dat=cr_pub%20%200pubmed
#	**Home Health Care - Finland**	Study survey on seamlessness of service and cooperation; flow of info between health and social care personnel; quality of services; available tools for information exchange; respondent health and working capacity	Eija G, Marja-Leena P. Home care personnel's perspectives on successful discharge of elderly clients from hospital to home setting. Scand J Caring Sci 2005;19:288-295.
#	**Hospital to Home Survey**	Assessment of organization initatives to reduce readmissions and partnering with community physicians or physician groups.	E. H. Bradley, L. Curry, L. I. Horwitz et al., "Hospital Strategies Associated with 30-Day Readmission Rates for Patients with Heart Failure," Circulation: Cardiovascular Quality and Outcomes, July 2013 6(4):444–50. https://www.ahajournals.org/action/downloadSupplement?doi=10.1161%2FCIRCOUTCOMES.111.000101&file=000101r1_b_evaluating_hospital_strategies_for_reducing_30_day_readmission_rates_b_.pdf
#	**Information Needs Survey**	Home-based care nurse survey regarding the information that geriatric patients do not have when they come home from the hospital, and how lacking that information influences whether or not they will be readmitted to the hospital	Romagnoli KM, Handler SM, Ligons FM, Hochheiser H. Home-care nurses' perceptions of unmet information needs and communication difficulties of older patients in the immediate post-hospital discharge period. BMJ Quality & Safety /20;22:324-332.
#	**Measure of Processes of Care for Service Providers**	Assess provider perceptions of the care they provide to children and the families, and their family-centered approach.	Dyke P, Buttigieg P, Blackmore AM, Ghose A. Use of the Measure of Process of Care for families (MPOC-56) and service providers (MPOC-SP) to evaluate family-centred services in a paediatric disability setting. Child: Care32:167-176.
#	**Modified Physician-PREPARED Scale**	Measure qualities of hospital discharge from the outpatient physician perspective including quality and outcome of discharge planning and communications	Graumlich JF, Grimmer-Somers K, Aldag JC. Discharge planning scale: community physicians' perspective. J Hosp Med 2008;3:455-464.
#	**Next Step Nursing Home Self Assessment Survey**	Nursing home self-assessment survey to help staff evaluate how well they work with family caregivers in planning transitions.	Nursing Home Performance Self-Assessment Survey. Next Step In Care. Accessed 2009. https://www.nextstepincare.org/uploads/File/Surveys/Nursing_Home/Nursing_Homes_Provider_Survey.pdf
#	**North Carolina Preventing Avoidable Readmissions Collaborative survey**	Guided questionnaire about about care coordination, information exchange, follow-up care, accountability, and medication management.	Jones CD, Vu MB, O'Donnell CM et al. A failure to communicate: a qualitative exploration of care coordination between hospitalists and primary care providers around patient hospitalizations. J Gen Intern Med 2015;30:417-424.
#	**Out of Sight, Out of Mind**	Physician perspectives on factors limiting the quality of discharge care. Themes identified included 1) competing priorities in the discharge process; (2) inadequate coordination within multidisciplinary discharge teams; (3) lack of standardization in discharge procedures; (4) poor patient and family communication; and (5) lack of postdischarge feedback and clinical responsibility.	Greysen SR, Schiliro D, Horwitz LI, Curry L, Bradley EH. "Out of sight, out of mind": housestaff perceptions of quality-limiting factors in discharge care at teaching hospitals. J Hosp Med 2012;7:376 381.

#	**Patient Activation Assessment Tool Guidelines**	A 10 item instrument to measure the level of activation of the patient at the beginning and the end of the intervention. The PAA aligned with the four pillars of the intervention, specifically, medication management, red flags, medical care follow-up and use of a personal health record.	Coleman, Eric. Patient Activation Assessment Guidelines. The Care Transitions Program. https://caretransitions.org/wp-content/uploads/2015/06/PAA_Tool_Guidelines.pdf
#	**Patient and Family Engagement Survey**	Highlights the extent of progress hospitals have made in engaging patients and their family members Areas discussed include 1.How the hospital partners with patient and family advisors, 2. Family presence (visitor) policies and practices, 3. Practices that help patients make decisions, 4.Practices used at the bedside, 5. Patient and Family training and education.	Herrin J, Harris KG, Kenward K, Hines S, Joshi MS, Frosch DL. Patient and family engagement: a survey of US hospital practices. BMJ Qual Saf. 2016 Mar;25(3):182-9. doi: 10.1136/bmjqs-2015-004006. Epub 2015 Jun 16. PMID: 26082560; PMCID: PMC4789699.
#	**Post-ICU Adverse Events Questionnaire**	Exploring clinicians' opinions of factors associated with post-Intensive Care adverse events, including the system/enviornement, human factors, and patient factors.	Elliott M, Page K, Worrallâ€Carter L, Rolley J. Examining adverse events after intensive care unit discharge: Outcomes from a pilot questionnaire. International Journal of Nursing Practice 2013;19:479-486.
#	**Queen's University Family Medicine Continuity of Care Interview**	To understand how the conception of continuity of care can influence family physician trainees by exploring the perspectives of established family physicians, physicians working in episodic care	Delva D, Kerr J, Schultz K. Continuity of care: Differing conceptions and values. Canadian Family Physician /20;57:915-921.
#	**Radboud University Nijmegen Medical Centre Questionnaire**	Questionnaires among stakeholders to assess: (i) how hospital discharge is experienced in daily practice and (ii) what is perceived to be important in the handover process at discharge.	Hesselink G, Schoonhoven L, Plas M, Wollersheim H, Vernooij-Dassen M. Quality and safety of hospital discharge: a study on experiences and perceptions of patients, relatives and care providers. Int J Qual Health Care 2013;25:66-74.
#	**Readiness for Hospital Discharge Scale**	Nurse and patient discharge readiness assessment forms.	Weiss ME, Costa LL, Yakusheva O, Bobay KL. Validation of patient and nurse short forms of the Readiness for Hospital Discharge Scale and their relationship to return to the hospital. Health Serv Res 2014;49:304-317
#	**Roles and Responsibilities of Clinicians Interview**	In-depth interviews to identify the perceived roles and responsibilities of clinicians during care transitions of older adults.	Schoenborn NL, Arbaje AI, Eubank KJ, Maynor K, Carrese JA. Clinician Roles and Responsibilities During Care Transitions of Older Adults. Journal of the American Geriatrics Society /20;61:231-236.
#	**Smart Family Foundation Discharge Survey**	Survey of physicians with an outpatient practice to evaluate satisfaction with current discharge summaries, perceptions of preventable adverse events related to suboptimal information transfer, and the perceived need for the electronic discharge summaries.	O'Leary KJ, Liebovitz DM, Feinglass J, Liss DT, Baker DW. Outpatient physicians' satisfaction with discharge summaries and perceived need for an electronic discharge summary. J Hosp Med 2006;1:317-320.
