# Supplementary material for: Development and psychometric properties of surveys to assess provider perspectives on the barriers and facilitators of effective care transitions
Source: BMC Health Serv Res. 2021 May 20;21:478. doi: 10.1186/s12913-021-06369-5 (PMC8136156; doi:10.1186/s12913-021-06369-5)
Supplement: Supplementary file 2 — Additional file 2. ACHIEVE Provider Survey. ACHIEVE Downstream Provider, Ambulatory Provider, and Hospital Provider Surveys to assess transitional care. A set of surveys to be administered to providers to learn about their work experiences delivering transitional care to patients. Developed by Westat, the Provider Surveys’ goal is to assess the barriers and facilitators in delivering transitional care services and describe the organizational and community contexts in providing transitional care services. [file 12913_2021_6369_MOESM2_ESM.pdf]

TITLE PAGE

Title: Development and psychometric properties of surveys to assess provider perspectives on the barriers and facilitators of effective care transitions

Supplement 2: ACHIEVE Downstream Provider, Ambulatory Provider and Hospital Provider Surveys to assess transitional care

Authors
Maurice C. Johnson, Jr., MPH[1]
Helen Liu, BS[1]
Joann Sorra, PhD[1]
Jane Brock, MD, MSPH[2]
Brianna Gass, MPH[2]
Jing Li, MD, DrPH, MS[3]
Jessica Miller Clouser, MPH[3]
Karen Hirschman, PhD, MSW[4]
Deborah Carpenter, RN, MSN[1]
Huong Q, Nguyen, PhD, RN[5]
Mark V. Williams, MD, MHM[3]

[1] Westat, Rockville, MD
[2] Telligen Quality Improvement Organization, Denver, CO
[3] Center for Health Services Research, University of Kentucky, Lexington, KY
[4] University of Pennsylvania, Philadelphia, PA
[5] Kaiser Permanente Southern California, Pasadena, CA

Corresponding Author
Maurice C. Johnson, MPH
Westat
1600 Research Boulevard, RB 1189
mauricejohnson@westat.com
240-453-2640

ACHIEVE Downstream Provider, Ambulatory Provider and Hospital Provider Surveys to assess transitional care

Background

The Patient-Centered Outcomes Research Institute (PCORI) funded Project ACHIEVE (Achieving Patient-Centered Care and Optimized Health in Care Transitions by Evaluating the Value of Evidence), which is a research study aimed at identifying the transitional care services and outcomes that matter most to patients and caregivers. Transitional care is the coordination and continuity of health care when a patient moves from one healthcare setting to another or back home. Led by the University of Kentucky, project ACHIEVE also evaluates the effectiveness of transitional care improvement efforts and develops recommendations of best practices for patient-centered care transitions, and guidance for spreading them across the U.S.

As part of the project, providers were asked about their work experiences in delivering transitional care to patients. Developed by Westat, the goal of the Provider Surveys is both to assess the barriers and facilitators in delivering transitional care services, and to describe the organizational and community contexts in delivering transitional care services.

There are three versions of the Provider Survey capturing different provider perspectives in providing transitional care services:

- **Downstream Provider:** This survey is health care providers in skilled nursing facilities, home health agencies, or other community-based organizations who coordinate with hospitals to provide care to patients recently discharged from a hospital. This includes intake coordinators, care coordinators, health coaches, or similar positions.
- **Ambulatory Provider:** This survey is for primary and specialty care providers in ambulatory care settings who coordinate with hospitals to provide care to patients recently discharged from a hospital. This includes physicians, physician assistants, and nurse practitioners who provide care to patients discharged from a pre-identified hospital.
- **Hospital Provider:** This survey is for hospital providers who spend much of their time helping with hospital discharges. This includes case managers, care coordinators, nurses, physicians NPs/PAs, pharmacists, and/or social workers who provide direct care/services to patients being discharged from a hospital.

For more information about the Provider Surveys, contact:

Mark V. Williams, MD, FACP, MHM
Director, Center for Health Services Research
University of Kentucky, Chief Transformation & Learning Officer
Phone: 859-218-1037
740 South Limestone
J525 Kentucky Clinic
Lexington, KY 40536-0284
Mark.Will@uky.edu
https://achieve.med.uky.edu/

Page Intentionally Blank

Overview of the Provider Surveys

The surveys are organized by one or more of the following sections:

1) **Information about Patients:*** Downstream and ambulatory providers report on when they are aware that a patient is admitted and discharged from a hospital. Providers also assess how comprehensive the information about a discharged patient is.
2) **Communication with Caregivers:** Providers report on challenges experienced when trying to engage family and friend caregivers and the strategies they used to try overcome those challenges.
3) **Health Information Technology:*** Downstream and ambulatory providers report on how they exchange patient information with a hospital, and their access to health information technology systems.
4) **Organization Support for Transitional Care:** Providers' perspectives on the organizational and senior leadership support for providing transitional care services.
5) **Access to Community Resources:** Provider perspectives on patient access to services and health-related resources within a community.
6) **Working with Other Providers**: Providers' perspectives on their relationship working with other types of providers in the community when providing transitional care to patients.
7) **Overall Coordination with Hospital:*** Downstream and ambulatory providers' overall assessment on how well hospital coordinates with the provider in delivering care to patients discharged from the hospital.
8) **Overall Assessment of Transitional Care:** Providers' overall assessment on how well its organization provides transitional care to patients.
9) **Provider Characteristics:** General provider characteristics. Each version of the survey has its own specified set of provider characteristic questions.

* Indicates section only within the Downstream Provider and Ambulatory Provider Surveys

The surveys consist of a series of single descriptive items, single item measures, composite measures, and overall ratings. Table 1 provides an overview of the survey items among the Downstream Provider, Ambulatory Provider, and Hospital Provider surveys.

Table 1. Final Composites and Single Items

Composites	Downstream Provider	Ambulatory Provider	Hospital Provider
Effort in Coordinating Patient Care (3 or 4 items)	Q6, Q7, Q8	Q6, Q7, Q8	Q6, Q7, Q8, Q10*
Quality of Patient Information Received (4 items)	Q11, Q12, Q13, Q14	Q11, Q12, Q13, Q14	Q11, Q12, Q13, Q14
Organizational Support for Transitional Care (2 or 3 items)	Q17, Q18, Q20*	Q17, Q18	Q17, Q18
Access to Community Resources (6 items)	Q21, Q22, Q23, Q24, Q25, Q26	Q21, Q22, Q23, Q24, Q25, Q26	Q21, Q22, Q23, Q24, Q25, Q26
Strength of Relationships Among Community Providers (4 items)	Q27, Q28, Q29, Q30	Q27, Q28, Q29, Q30	Q27, Q28, Q29, Q30
**Single Item Measures**			
When are you typically made aware that a patient was admitted to hospital? (R)	Q2	Q2	--
When are you typically made aware that a patient was discharged from hospital? (R)	Q4	Q4	--
For how many recently discharged patients do you typically receive a discharge summary?	Q5	Q5	--
How many patients have a family or friend caregiver with who you interact?	Q15	Q15	Q15
Too many of the patients referred to our services have more acute conditions than we are able to handle. (R)	Q9*	--	--
Reducing hospital readmissions for patients is a priority in my organization	Q19	Q19	Q19
**Single Items (Descriptive)**			
How do you typically know that a patient was admitted to hospital?	Q1	Q1	--
How do you typically know that a patient was discharged from hospital?	Q3	Q3	--
Do you have access to hospital's health information technology system to get information about patients?	Q16	Q16	--
Provider characteristics questions	PC-D1, PC-D2, PC-D3, PC-D4	PC-A1, PC-A2, PC-A3, PC-A4, PC-A5, PC-A6	PC-H1, PC-H2, PC-H3
**Overall Ratings**			
Please rate how well the hospital coordinates with you when working with recently discharged patients.	Q31	Q31	--
Please rate how well your organization helps transition patients from the hospital to another healthcare setting or back home.	Q32	Q32	Q32

(R) Indicates that the item responses were recoded so that the higher scores represent more positive responses.
* Indicates that the item is unique to the specific survey.

The surveys are intended to solicit feedback from providers about their interactions with a specific hospital. Each survey is customized with the name of the hospital the provider interacts or is affiliated with, in the designated areas that indicate **[insert HOSPITAL NAME]**. In addition, items that are unique to a specific version of survey are marked as the following: D – Downstream, A – Ambulatory, H – Hospital.

ACHIEVE Provider Survey

COORDINATION OF TRANSITIONAL CARE SURVEY

Thank you for your participation in this survey, which asks about your work in transitional care. Transitional care is the coordination and continuity of health care when a patient moves from one healthcare setting to another or back home. You will be asked about the transitional care activities you are involved in that assist patients transitioning from a hospital to another care facility or back home. Other questions will ask about your experience coordinating with other providers in your community.

SURVEY ELIGIBILITY

Do you provide care or services to patients who are discharged from [insert HOSPITAL NAME]? (D,A)

☐1 Yes

☐2 No

Is your primary place of work in [insert HOSPITAL NAME] or on its campus? (H)

☐1 Yes

☐2 No

I. INFORMATION ABOUT PATIENTS

1. How do you typically know that a patient was admitted to [insert HOSPITAL NAME]? Choose one. (D,A)

- ☐1 The hospital informs me
- ☐2 I proactively look for this information
- ☐3 The patient or patient's family/friend informs me
- ☐4 Some other way
- ☐5 I typically do not know that a patient was admitted [GO TO Question 3]

2. When are you typically made aware that a patient was admitted to [insert HOSPITAL NAME]? Choose one. (D,A)

- ☐1 Within 24 hours after a patient is admitted
- ☐2 More than 24 hours after a patient is admitted but is still in the hospital
- ☐3 The same day a patient is being discharged
- ☐4 Between 1 and 3 days after a patient is discharged
- ☐5 More than 3 days after a patient is discharged

3. How do you typically know that a patient was discharged from [insert HOSPITAL NAME]? Choose one. (D,A)

- ☐1 The hospital informs me
- ☐2 I proactively look for this information
- ☐3 The patient or patient's family/friend informs me
- ☐4 Some other way
- ☐5 I typically do not know that a patient was discharged [GO TO Question 5]

4. When are you typically made aware that a patient was discharged from [insert HOSPITAL NAME]? Choose one. (D,A)

- ☐1 The day before a patient is discharged
- ☐2 The same day a patient is being discharged
- ☐3 Between 1 and 3 days after a patient is discharged
- ☐4 More than 3 days after a patient is discharged

5. Thinking about patients recently discharged from [insert HOSPITAL NAME], for how many do you typically receive a discharge summary? (D,A)

- ☐1 None or very few
- ☐2 Less than half
- ☐3 About half
- ☐4 More than half
- ☐5 All or almost all
- ☐6 Don't know

To what extent do you agree or disagree with the following statements?<br>Working with [insert HOSPITAL NAME]… (D,A)<br>Within your hospital… (H)	Strongly Disagree	Disagree	Neither agree or disagree	Agree	Strongly Agree	Does not apply or don't know
**6.** It is easy to get information about a recently discharged patient.	☐1	☐2	☐3	☐4	☐5	☐6
**7.** It is easy to connect with providers and staff in the hospital to discuss a patient's care.	☐1	☐2	☐3	☐4	☐5	☐6
**8.** It is clear what in-patient procedures and tests have been performed and the results.	☐1	☐2	☐3	☐4	☐5	☐6
**9.** Too many of the patients referred to our services have more acute conditions than we are able to handle. (D)	☐1	☐2	☐3	☐4	☐5	☐6
**10.** Everyone involved in the patient's care understands what needs to be done for the patient. (H)	☐1	☐2	☐3	☐4	☐5	☐6

For patients recently discharged from [insert HOSPITAL NAME], how often is the information you receive… (D,A)<br>Within your hospital, how often is the information you receive about patients… (H)	Never	Rarely	Sometimes	Usually	Always	Does not apply or don't know
**11.** In a format where it is easy to find important information?	☐1	☐2	☐3	☐4	☐5	☐6
**12.** Complete?	☐1	☐2	☐3	☐4	☐5	☐6
**13.** Available as soon as it is needed?	☐1	☐2	☐3	☐4	☐5	☐6
**14.** Clear about who to follow up with at the hospital if you have questions or concerns about the patient?	☐1	☐2	☐3	☐4	☐5	☐6

II. COMMUNICATION WITH CAREGIVERS

15. When working with patients discharged from the hospital, about how many of them have a family or friend caregiver with whom you interact?

☐1 None or very few
☐2 Less than half
☐3 About half
☐4 More than half
☐5 All or almost all
☐6 Don't know

III. HEALTH INFORMATION TECHNOLOGY

16. Do you have access to [insert HOSPITAL NAME]'s health information technology system to review information about patients? (D,A)

☐1 Yes
☐2 No

IV. MY ORGANIZATION'S SUPPORT FOR TRANSITIONAL CARE

Transitional care is the coordination of health care when a patient moves from one healthcare setting to another or back home.

How much do you agree or disagree with the following statements?	Strongly Disagree ▼	Disagree ▼	Neither agree or disagree ▼	Agree ▼	Strongly Agree ▼	Does not apply or don't know ▼
**17.** My organization is implementing activities to improve transitional care for patients.	☐1	☐2	☐3	☐4	☐5	☐6
**18.** Senior leaders in my organization dedicate adequate resources to support effective transitional care for patients.	☐1	☐2	☐3	☐4	☐5	☐6
**19.** Reducing hospital readmissions for patients is a priority in my organization.	☐1	☐2	☐3	☐4	☐5	☐6
**20.** My organization tries to increase physician awareness and understanding of the services we provide that can assist recently discharged patients. (D)	☐1	☐2	☐3	☐4	☐5	☐6

V. ACCESS TO COMMUNITY RESOURCES

How much do you agree or disagree with the following statements?

In the local area your organization serves, patients have adequate access to…	Strongly Disagree ▼	Disagree ▼	Neither agree or disagree ▼	Agree ▼	Strongly Agree ▼	Does not apply or don't know ▼
**21.** Primary care providers.	☐1	☐2	☐3	☐4	☐5	☐6
**22.** Specialty providers.	☐1	☐2	☐3	☐4	☐5	☐6
**23.** Skilled nursing and rehabilitation facilities.	☐1	☐2	☐3	☐4	☐5	☐6
**24.** Mental health/behavioral health services.	☐1	☐2	☐3	☐4	☐5	☐6
**25.** In-home support services (e.g., home health aides/technicians or other services that help patients remain in their homes).	☐1	☐2	☐3	☐4	☐5	☐6
**26.** Transportation for medical related services.	☐1	☐2	☐3	☐4	☐5	☐6

VI. WORKING WITH OTHER PROVIDERS

How would you describe the relationship between you and the following providers in working together to provide transitional care to patients?	Poor ▼	Fair ▼	Good ▼	Very Good ▼	Excellent ▼	Don't work with this type of provider ▼
**27.** Primary care providers and specialists.	☐1	☐2	☐3	☐4	☐5	☐6
**28.** Skilled nursing and rehabilitation facilities.	☐1	☐2	☐3	☐4	☐5	☐6
**29.** Home health agencies.	☐1	☐2	☐3	☐4	☐5	☐6
**30.** Community-based organizations.	☐1	☐2	☐3	☐4	☐5	☐6

VII. OVERALL COORDINATION WITH HOSPITAL

31. Please rate how well [insert HOSPITAL NAME] coordinates with you when working with recently discharged patients: (D,A)

☐1 Poor
☐2 Fair
☐3 Good
☐4 Very Good
☐5 Excellent

VIII. OVERALL ASSESSMENT OF TRANSITIONAL CARE

32. Please rate how well your organization helps transition patients from the hospital to another healthcare setting or back home:

☐1 Poor
☐2 Fair
☐3 Good
☐4 Very Good
☐5 Excellent

IX. PROVIDER CHARACTERISTICS (DOWNSTREAM)

PC-D1. Which type of organization best describes your primary place of work? Choose one.

- ☐1 Skilled nursing facility
- ☐2 Home health agency
- ☐3 Area agency on aging
- ☐4 Community-based organization
- ☐5 Other

PC-D2. What position do you hold within your organization? Select ONE answer that best describes your position.

- ☐1 Administrator/Manager
- ☐2 Primary care physician
- ☐3 Specialty care physician
- ☐4 Physician assistant
- ☐5 Nurse practitioner/Advanced practice nurse
- ☐6 Nurse
- ☐7 Nurse assistant
- ☐8 Pharmacist or other pharmacy staff
- ☐9 Social worker
- ☐10 Case manager/Care manager
- ☐11 Care coordinator
- ☐12 Patient navigator/Care navigator
- ☐13 Coach for care transitions
- ☐14 Patient educator
- ☐15 Intake/Enrollment coordinator
- ☐16 Discharge coordinator
- ☐17 Community health worker
- ☐18 Other

**As part of your job…**	**Yes** ▼	**No** ▼
**PC-D3.** Do you provide clinical care to patients?	☐1	☐2
**PC-D4.** Do you perform or review the results of assessments to identify patients at risk of readmission?	☐1	☐2

IX. PROVIDER CHARACTERISTICS (AMBULATORY)

PC-A1. You are a...

- ☐1 Primary care physician
- ☐2 Specialty care physician
- ☐3 Physician assistant
- ☐4 Nurse practitioner/Advanced practice nurse
- ☐5 Other

PC-A2. Which best describes the majority ownership of your practice?

- ☐1 Provider(s) and/or Physician(s)
- ☐2 Hospital or Health System
- ☐3 University or Academic Medical Center
- ☐4 Community Health Center
- ☐5 Federal, state, or local government
- ☐6 Other

PC-A3. Including yourself, how many providers work in your practice? A provider is a physician, physician assistant, or nurse practitioner/advanced practice nurse?

- ☐1 One, I am a solo practitioner
- ☐2 2-5 providers
- ☐3 6-10 providers
- ☐4 More than 10 providers

PC-A4. Which of the following best describes your practice?

- ☐1 Single specialty-Primary care
- ☐2 Single specialty-NOT primary care
- ☐3 Multispecialty that includes primary care
- ☐4 Multispecialty that DOES NOT include primary care

PC-A5. Is your practice currently participating in a patient-centered medical home (PCMH) program?

- ☐1 Yes
- ☐2 No
- ☐3 Don't know

PC-A6. Is your practice part of an Accountable Care Organization (either a Medicare or Commercial ACO) or in the process of becoming one? (A)

- ☐1 Yes
- ☐2 No
- ☐3 Don't know

PROVIDER CHARACTERISTICS (HOSPITAL)

PC-H1. What position do you hold within your organization? Select ONE answer that best describes your position.

- ☐1 Administrator/Manager
- ☐2 Primary care physician
- ☐3 Specialty care physician
- ☐4 Physician assistant
- ☐5 Nurse practitioner/Advanced practice nurse
- ☐6 Nurse
- ☐7 Nurse assistant
- ☐8 Pharmacist or other pharmacy staff
- ☐9 Social worker
- ☐10 Case manager/Care manager
- ☐11 Care coordinator
- ☐12 Patient navigator/Care navigator
- ☐13 Coach for care transitions
- ☐14 Patient educator
- ☐15 Intake/Enrollment coordinator
- ☐16 Discharge coordinator
- ☐17 Community health worker
- ☐18 Other

As part of your job…	Yes ▼	No ▼
**PC-H2.** Do you provide clinical care to patients?	☐1	☐2
**PC-H3.** Do you perform or review the results of assessments to identify patients at risk of readmission?	☐1	☐2

END OF SURVEY
THANK YOU
