# Supplementary material for: Development and psychometric properties of surveys to assess provider perspectives on the barriers and facilitators of effective care transitions
Source: BMC Health Serv Res. 2021 May 20;21:478. doi: 10.1186/s12913-021-06369-5 (PMC8136156; doi:10.1186/s12913-021-06369-5)
Supplement: Supplementary file 3 — Additional file 3. Summary of Responses Table. Summary of Responses Table-Mean, Standard Deviation, Percent Positive, Missing, Does Not Apply/Don’t Know Results. The table includes the mean, standard deviation, percent positive, missing, does not apply/don’t know results of each survey item by provider type. [file 12913_2021_6369_MOESM3_ESM.pdf]

TITLE PAGE

Title: Development and psychometric properties of surveys to assess provider perspectives on the barriers and facilitators of effective care transitions

Supplement 3: Summary of Responses Table-Mean, Standard Deviation, Percent Positive, Missing, Does Not Apply/ Don't Know Results

Authors
Maurice C. Johnson, Jr., MPH[1]
Helen Liu, BS[1]
Joann Sorra, PhD[1]
Jane Brock, MD, MSPH[2]
Brianna Gass, MPH[2]
Jing Li, MD, DrPH, MS[3]
Jessica Miller Clouser, MPH[3]
Karen Hirschman, PhD, MSW[4]
Deborah Carpenter, RN, MSN[1]
Huong Q, Nguyen, PhD, RN[5]
Mark V. Williams, MD, MHM[3]

[1] Westat, Rockville, MD
[2] Telligen Quality Improvement Organization, Denver, CO
[3] Center for Health Services Research, University of Kentucky, Lexington, KY
[4] University of Pennsylvania, Philadelphia, PA
[5] Kaiser Permanente Southern California, Pasadena, CA

Corresponding Author
Maurice C. Johnson, MPH
Westat
1600 Research Boulevard, RB 1189
mauricejohnson@westat.com
240-453-2640

Summary of Responses Table-Mean, Standard Deviation, Percent Positive, Missing, Does Not Apply/ Don't Know Results

Survey Items		Provider Type	Mean	Std Dev	% Positive	% MI/NA/DK
Q1	How do you typically know that a patient was admitted to hospital? (descriptive)	Downstream	--	--	--	0%
		Ambulatory	--	--	--	0%
		Hospital	--	--	--	--
Q2	When are you typically made aware that a patient was admitted to hospital? (admission scale)	Downstream	4.19	1.09	80%	0%
		Ambulatory	4.32	1.13	81%	<1%
		Hospital	--	--	--	--
Q3	How do you typically know that a patient was discharged from hospital? (descriptive)	Downstream	--	--	--	1%
		Ambulatory	--	--	--	0%
		Hospital	--	--	--	--
Q4	When are you typically made aware that a patient was discharged from hospital? (discharge scale)	Downstream	3.01	0.79	76%	0%
		Ambulatory	2.71	0.70	66%	<1%
		Hospital	--	--	--	--
Q5	For how many recently discharged patients do you typically receive a discharge summary? (N/LH/AH/MH/A/DK)	Downstream	4.08	1.48	75%	5%
		Ambulatory	3.61	1.68	64%	5%
		Hospital	--	--	--	--
Q6	It is easy to get information about a recently discharged patient. (SD/D/N/A/SA/DK)	Downstream	4.12	1.03	82%	3%
		Ambulatory	4.18	1.01	84%	<1%
		Hospital	4.09	1.01	81%	2%
Q7	It is easy to connect with providers and staff in the hospital to discuss a patient's care. (SD/D/N/A/SA/DK)	Downstream	3.69	1.16	67%	4%
		Ambulatory	3.40	1.20	53%	5%
		Hospital	4.06	0.95	81%	1%
Q8	It is clear what in-patient procedures and tests have been performed and the results. (SD/D/N/A/SA/DK)	Downstream	3.98	1.00	76%	4%
		Ambulatory	3.91	1.03	77%	1%
		Hospital	4.11	0.97	84%	3%
Q9	Too many of the patients referred to our services have more acute conditions than we are able to handle. (SD/D/N/A/SA/DK)	Downstream	3.68	0.97	62%	4%
		Ambulatory	--	--	--	--
		Hospital	--	--	--	--
Q10	Everyone involved in the patient's care understands what needs to be done for the patient. (SD/D/N/A/SA/DK)	Downstream	--	--	--	--
		Ambulatory	--	--	--	--
		Hospital	3.54	1.02	57%	1%
Q11		Downstream	4.10	0.79	80%	3%

Survey Items		Provider Type	Mean	Std Dev	% Positive	% MI/NA/DK
	For recently discharged patients, how often is the information you receive: In a format where it is easy to find important information? (N/R/S/U/A/DK)	Ambulatory<br>Hospital	3.93<br>4.04	0.91<br>0.68	73%<br>83%	1%<br>1%
Q12	For recently discharged patients, how often is the information you receive: Complete? (N/R/S/U/A/DK)	Downstream<br>Ambulatory<br>Hospital	3.96<br>3.87<br>3.87	0.73<br>0.84<br>0.68	80%<br>73%<br>74%	4%<br>1%<br>1%
Q13	For recently discharged patients, how often is the information you receive: Available as soon as it is needed? (N/R/S/U/A/DK)	Downstream<br>Ambulatory<br>Hospital	3.90<br>3.87<br>3.80	0.77<br>0.85<br>0.69	72%<br>74%<br>70%	4%<br>3%<br>2%
Q14	For recently discharged patients, how often is the information you receive: Clear about who to follow up with at the hospital if you have questions or concerns about the patient? (N/R/S/U/A/DK)	Downstream<br>Ambulatory<br>Hospital	3.80<br>3.50<br>3.83	1.00<br>1.09<br>0.83	67%<br>53%<br>68%	4%<br>2%<br>2%
Q15	How many patients have a family or friend caregiver with whom you interact? (N/LH/AH/MH/A/DK)	Downstream<br>Ambulatory<br>Hospital	3.91<br>3.39<br>3.52	1.08<br>1.11<br>1.05	72%<br>48%<br>57%	3%<br>3%<br>7%
Q16	Do you have access to the hospital's health information technology system to get information about patients? (Y/N)	Downstream<br>Ambulatory<br>Hospital	--<br>--<br>--	--<br>--<br>--	--<br>--<br>--	<1%<br><1%<br>--
Q17	My organization is implementing activities to improve transitional care for patients. (SD/D/N/A/SA/DK)	Downstream<br>Ambulatory<br>Hospital	4.56<br>4.24<br>4.36	0.84<br>1.06<br>0.77	**92%**<br>83%<br>88%	2%<br>5%<br>2%
Q18	Senior leaders in my organization dedicate adequate resources to support effective transitional care for patients. (SD/D/N/A/SA/DK)	Downstream<br>Ambulatory<br>Hospital	4.28<br>3.87<br>3.72	1.00<br>1.17<br>1.14	84%<br>71%<br>66%	4%<br>5%<br>2%
Q19	Reducing hospital readmissions for patients is a priority in my organization. (SD/D/N/A/SA/DK)	Downstream<br>Ambulatory<br>Hospital	4.71<br>4.53<br>4.53	0.77<br>0.86<br>0.78	**95%**<br>**93%**<br>**91%**	2%<br>3%<br>1%
Q20	My organization tries to increase physician awareness and understanding of the services we	Downstream<br>Ambulatory<br>Hospital	4.38<br>--<br>--	0.91<br>--<br>--	88%<br>--<br>--	3%<br>--<br>--

	Survey Items	Provider Type	Mean	Std Dev	% Positive	% MI/NA/DK
	provide that can assist recently discharged patients. (SD/D/N/A/SA/DK)					
Q21	In the local area your organization serves, patients have adequate access to: Primary care providers. (SD/D/N/A/SA/DK)	Downstream<br>Ambulatory<br>Hospital	4.16<br>3.90<br>3.79	0.90<br>1.13<br>1.05	86%<br>77%<br>73%	2%<br>2%<br>1%
Q22	In the local area your organization serves, patients have adequate access to: Specialty providers. (SD/D/N/A/SA/DK)	Downstream<br>Ambulatory<br>Hospital	4.02<br>3.89<br>3.65	0.99<br>1.05<br>1.10	80%<br>78%<br>66%	3%<br>4%<br>2%
Q23	In the local area your organization serves, patients have adequate access to: Skilled nursing and rehabilitation facilities. (SD/D/N/A/SA/DK)	Downstream<br>Ambulatory<br>Hospital	4.25<br>3.85<br>3.80	0.90<br>1.00<br>0.99	86%<br>73%<br>74%	5%<br>6%<br>3%
Q24	In the local area your organization serves, patients have adequate access to: Mental health/behavioral health services. (SD/D/N/A/SA/DK)	Downstream<br>Ambulatory<br>Hospital	3.15<br>2.70<br>2.72	1.29<br>1.32<br>1.27	49%<br>32%<br>34%	3%<br>5%<br>2%
Q25	In the local area your organization serves, patients have adequate access to: In-home support services (e.g., home health aides/ technicians or other services that help patients remain in their homes). (SD/D/N/A/SA/DK)	Downstream<br>Ambulatory<br>Hospital	3.92<br>3.54<br>3.64	1.05<br>1.09<br>0.99	76%<br>60%<br>68%	5%<br>6%<br>4%
Q26	In the local area your organization serves, patients have adequate access to: Transportation for medical related services. (SD/D/N/A/SA/DK)	Downstream<br>Ambulatory<br>Hospital	3.44<br>3.35<br>3.13	1.16<br>1.05<br>1.16	59%<br>51%<br>46%	5%<br>6%<br>5%
Q27	How would you describe the relationship between you and the following providers in working together to provide transitional care to patients? Primary care providers and specialists. (P/F/G/VG/E/D)	Downstream<br>Ambulatory<br>Hospital	3.84<br>3.78<br>3.51	0.99<br>1.01<br>1.10	64%<br>61%<br>55%	1%<br>2%<br>1%
Q28	How would you describe the relationship between you and the following providers in working together to provide transitional care to patients? Skilled nursing and rehabilitation facilities. (P/F/G/VG/E/D)	Downstream<br>Ambulatory<br>Hospital	3.88<br>2.98<br>3.33	1.00<br>1.17<br>1.10	67%<br>35%<br>44%	1%<br>2%<br>1%

	Survey Items	Provider Type	Mean	Std Dev	% Positive	% MI/NA/DK
Q29	How would you describe the relationship between you and the following providers in working together to provide transitional care to patients? Home health agencies. (P/F/G/VG/E/D)	Downstream<br>Ambulatory<br>Hospital	3.84<br>3.18<br>3.34	1.04<br>1.10<br>1.14	67%<br>40%<br>46%	1%<br>2%<br>2%
Q30	How would you describe the relationship between you and the following providers in working together to provide transitional care to patients? Community-based organizations. (P/F/G/VG/E/D)	Downstream<br>Ambulatory<br>Hospital	3.53<br>2.82<br>2.86	1.08<br>1.09<br>1.12	55%<br>27%<br>29%	2%<br>2%<br>2%
Q31	Please rate how well the hospital coordinates with you when working with recently discharged patients. (P/F/G/VG/E)	Downstream<br>Ambulatory<br>Hospital	3.48<br>3.16<br>--	0.99<br>1.07<br>--	49%<br>43%<br>--	2%<br>1%<br>--
Q32	Please rate how well your organization helps transition patients from the hospital to another healthcare setting or back home. (P/F/G/VG/E)	Downstream<br>Ambulatory<br>Hospital	3.89<br>3.44<br>3.44	0.87<br>1.02<br>0.95	69%<br>51%<br>51%	2%<br>1%<br>1%

Note: Mean scores are calculated such that higher scores represent more positive responses.

Admission scale – Within 24 hours after a patient is admitted/More than 24 hours after a patient is admitted but is still in the hospital/The same day a patient is being discharged/Between 1 and 3 days after a patient is discharged/More than 3 days after a patient is discharged

Discharge scale – The day before a patient is discharged/The same day a patient is being discharged/Between 1 and 3 days after a patient is discharged/More than 3 days after a patient is discharged

N/R/S/U/A/DK – Never/Rarely/Sometimes/Usually/Always/Does not apply or don't know

N/LH/AH/MH/A/DK – None or very few/Less than half/About half/More than half/All or almost all/Don't know

SD/D/N/A/SA/DK – Strongly disagree/Disagree/Neither agree or disagree/Agree/Strongly agree/Does not apply or don't know

P/F/G/VG/E/D – Poor/Fair/Good/Very good/Excellent/Don't work with this type of provider

P/F/G/VG/E – Poor/Fair/Good/Very good/Excellent

Y/N – Yes/No
